# The Effect of Desflurane on Neuronal Communication at a Central Synapse

**DOI:** 10.1371/journal.pone.0123534

**Published:** 2015-04-07

**Authors:** Jonathan Mapelli, Daniela Gandolfi, Enrico Giuliani, Francesco P. Prencipe, Federica Pellati, Alberto Barbieri, Egidio D’Angelo, Albertino Bigiani

**Affiliations:** 1 Dipartimento di Scienze Biomediche, Metaboliche e Neuroscienze, Università di Modena e Reggio Emilia, Modena, Italy; 2 Dipartimento di Scienze del Sistema Nervoso e del Comportamento, Università di Pavia, Pavia, Italy; 3 Dipartimento di Medicina Diagnostica, Clinica e di Sanità Pubblica, Università di Modena e Reggio Emilia, Modena, Modena, Italy; 4 Dipartimento di Scienze della Vita, Università di Modena e Reggio Emilia, Modena, Italy; 5 Brain Connectivity Center, C. Mondino National Neurological Institute, Pavia, Italy; McGill University, CANADA

## Abstract

Although general anesthetics are thought to modify critical neuronal functions, their impact on neuronal communication has been poorly examined. We have investigated the effect induced by desflurane, a clinically used general anesthetic, on information transfer at the synapse between mossy fibers and granule cells of cerebellum, where this analysis can be carried out extensively. Mutual information values were assessed by measuring the variability of postsynaptic output in relationship to the variability of a given set of presynaptic inputs. Desflurane synchronized granule cell firing and reduced mutual information in response to physiologically relevant mossy fibers patterns. The decrease in spike variability was due to an increased postsynaptic membrane excitability, which made granule cells more prone to elicit action potentials, and to a strengthened synaptic inhibition, which markedly hampered membrane depolarization. These concomitant actions on granule cells firing indicate that desflurane re-shapes the transfer of information between neurons by providing a less informative neurotransmission rather than completely silencing neuronal activity.

## Introduction

Halogenated anesthetics, by interacting with specific membrane proteins, affect synaptic transmission, membrane potential and signaling in neurons [1–3]. Desflurane, as other halogenated compounds, interacts with GABA-A receptors [4] and its binding site has been recently shown [5]. Moreover, desflurane regulates potassium [6] and sodium channel gating [7]. However, how the interaction of anesthetics with molecular targets affects information transfer among neurons has never been investigated in detail.

The language employed by neurons to communicate can be deciphered by using parameters taken from information and communication theory [8]. Among these parameters, mutual information (MI) was adapted to Neuroscience for quantifying the amount of information transmitted by single synapses [9], single neurons [10,11] or by large neuronal populations [12], therefore allowing the analysis of neural codes. MI descends directly from response entropy and noise entropy [13], which are correlated to the variability of responses to separate inputs [13] or to the same input [14–16], respectively. MI calculation provides a way to evaluate the capability of a neuronal system to separate different inputs and therefore to transmit information [17,18].

The cerebellar cortical circuit is an optimal preparation to calculate MI by virtue of a reduced input-output combination. In response to mossy fibers (mf) inputs, cerebellar (GrCs) respond with stereotyped patterns displaying a limited number of spikes (typically two or less [19,20]) which are confined in a restricted time window, by the intervention of Golgi cells inhibition [21,22]. Furthermore, although the presence of non-triggered spikes could contribute to increase response variability, spontaneous firing of GrCs in acute slices has never been observed. This peculiarity of GrCs response patterns lead to a low output variability [23] which, in turn, greatly reduces the complexity of calculations and the duration of recording sessions. The cerebellar circuitry can thus be used as a model to investigate the alterations induced by general anesthetics on neuronal circuit functioning. In addition, the interaction of general anesthetics with cerebellum has been poorly examined albeit several reports show functional changes of cerebellar activity during anesthesia [24–27].

In the present work, we have investigated how desflurane affects the information transfer between mf and GrCs in rat cerebellar slices. We experimentally evaluated MI at this synaptic stage by recording with the patch-clamp technique the GrCs responses to defined patterns of mf input signals. We found that desflurane modified spike generation patterns in GrCs leading to a substantial decrease of the mf-GrC MI, providing the first evidence of the effect of a clinically relevant anesthetic on information transfer in an intact neuronal circuit.

## Materials and Methods

Experiments were performed by using Sprague-Dawley rats at postnatal day P17-P24 [internal breeding, Charles-Rivers (Calco, Lecco, Italy)]. All experiments were conducted in accordance with international guidelines from the European Community Council Directive 86/609/EEC on the ethical use of animals. Experiments were approved by the Italian Minister of Health and by the Ethical Committee of the University of Modena and Reggio Emilia.

### Cerebellar Slices

Parasagittal cerebellar slices were obtained as described in [28]. Briefly, rats were anesthetized with isoflurane (Sigma-Aldrich, Saint Louis, MO, USA) and decapitated. The cerebellum was removed, the vermis isolated and fixed on a vibroslicer stage (VT1000S, Leica Microsystems, Nussloch, Germany) with cyanoacrylic glue. Acute 200-μm thick slices were cut in cold cutting solution containing (in mM): 130 K-gluconate, 15 KCl, 0.2 EGTA, 20 HEPES and 10 glucose, pH adjusted at 7.4 with NaOH. Slices were incubated at 32°C for at least 1 hour before recordings in oxygenated extracellular Krebs solution containing (in mM): 120 NaCl, 2 KCl, 1.2 MgSO_4_, 26 NaHCO_3_, 1.2 KH_2_PO_4_, 2 CaCl_2_, 11 glucose (pH 7.4 when equilibrated with 95% O_2_ and 5% CO_2_). Slices were then transferred to a recording chamber on the stage of an upright microscope (Zeiss Axioexaminer A1, Oberkochen, Germany) and perfused at 1.5 ml min^−1^ with oxygenated Krebs solution maintained at 32°C with a thermostatic controller (Multichannel system, Gmbh, Reuntlingen, Germany). Slices were immobilized with a nylon mesh attached to a platinum Ω-wire.

### Patch-clamp Recordings

Whole-cell recordings from GrCs were obtained with patch-clamp technique [20,29,30] by using an Axopatch 200B amplifier (Molecular Devices, Union City, CA, USA) (-3dB; cut-off frequency = 2 kHz). Recordings were digitized at 20 kHz using pClamp 9 (Molecular Devices) and a Digidata 1322A A/D converter (Molecular Devices). Patch pipettes were made with a vertical puller (model PP-830, Narishige, Tokyo, Japan) from borosilicate glass capillaries and filled with the following solution (in mM): 126 K-gluconate, 8 NaCl, 15 glucose, 5 HEPES, 1 MgSO_4_, 0.1 BAPTA-4K, 0.05 BAPTA-Ca^2+^, 3 ATP, 100 μM GTP; pH adjusted to 7.2 with KOH. This solution maintained resting free-[Ca^2+^] at 100 nM and pipettes had a resistance of 7–10 MΩ before seal formation.

**Fig 1:**
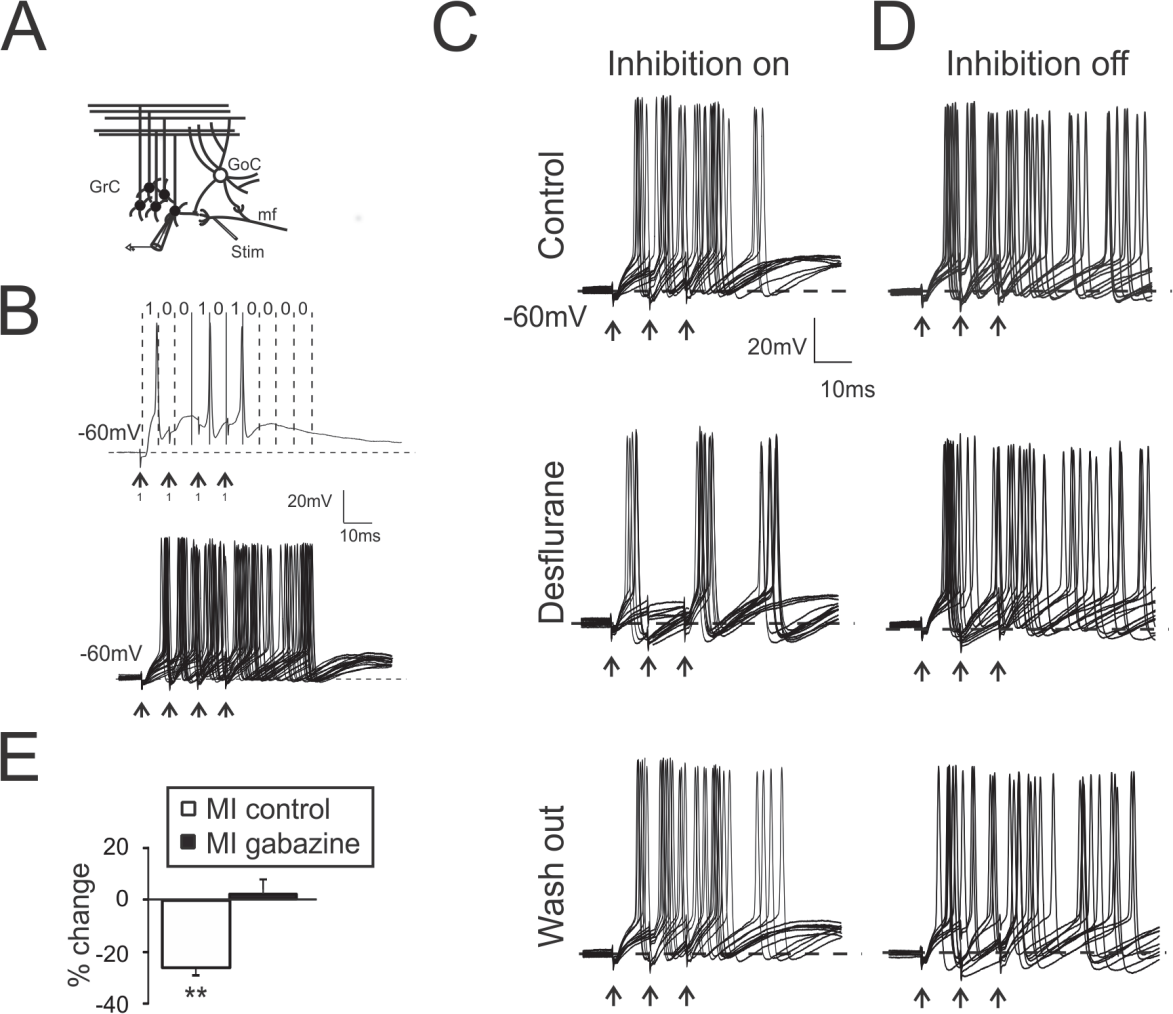
Desflurane modulates the MI between mf and GrCs. **A.** Scheme of the granular layer microcircuit. The stimulating electrode (Stim) was positioned onto the mossy fiber bundle (mf). GoC, Golgi cell; GrC, granule cell. **B.** Spike detection procedure generating binary digits. *Top* Single response of a GrC to 4 pulses at 100 Hz (mf code 1111, arrows). The spike in each time window determines the binary output (code 1001010000). *Bottom* Single GrC variability in response to the repetition of the same input pattern (25 repetition). **C.** Recordings (10 superimposed responses) from GrC following a 3-pulse, 100-Hz burst (mf code 1110, arrows) in control condition (*top*), during the application of desflurane (*middle*) and after wash-out (*bottom*). Desflurane (middle) decreases the total number of emitted spikes as well the the 1^st^ spike delay variability. The initial condition is fully recovered after anesthetic wash out (right). **D.** Recordings (10 superimposed responses) performed in the presence of gabazine. Note the increased number of spikes. GrC was stimulated with a 3-pulse, 100-Hz burst (mf code 1110, arrows). **E.** Histogram resumes MI changes induced by desflurane in control condition (n = 7) and in the presence of gabazine (n = 6). In this and in the following figures: * p < 0.05; ** p < 0.01.

Excitatory mossy fibers (Fig 1A) were stimulated by positioning a bipolar tungsten (Clark Instruments, Pangbourne, UK) electrode across the mossy fibers bundle. Stimulation intensity (± 5–15 V; 100 μs; via a stimulation isolation unit) was raised until excitatory synaptic activity generated at least 1 spike in GrCs at a membrane potential between -55 and -65 mV (mean -60.1 ± 1.3 n = 16). From a comparison with previous data [31] and mathematical models [30], in these conditions from 2 to 4 mossy fibers were stimulated per GrC depending on the level of synaptic inhibition. Excitatory post-synaptic potentials (EPSPs) were analyzed in terms of rise time, amplitude and total depolarization calculated as the integral of membrane depolarization between the onset and 50 ms from synaptic stimulation. Total depolarization was used as an index of membrane depolarization changes [32].

Axon bundles of the inhibitory Golgi cell were stimulated by positioning bipolar tungsten electrode in the granular layer. One or two stimuli at 50 Hz repeated at 0.1 Hz were used. Inhibitory post-synaptic currents (IPSCs) were detected in voltage-clamp configuration by holding neurons at 0 mV, and appeared as positive deflections given that chloride reversal potential was set at about -60 mV in our experimental conditions. IPSCs were isolated by adding to the bath solution 10 μM NBQX (Tocris Bioscience, Bristol, UK) and 25 μM D-APV (Tocris Bioscience, Bristol, UK) to block glutamate AMPA and NMDA receptors, respectively. NMDA currents were isolated by voltage clamping GrCs at -40 mV and in the presence of 10 μM SR9519 (Gabazine; Tocris Bioscience, Bristol UK), a selective GABA-A receptor blocker, and 10 μM NBQX. Peak amplitude, time to peak, rise time from 10 to 90% of peak amplitude (rise_10–90_) were computed. Decay components of synaptic currents were evaluated by calculating time constant (τ) of the mono-exponential fitting between the peak and the baseline. Total charge transfer was calculated by measuring IPSCs area [33]. The paired-pulse ratio (PPR) between first and second IPSC (PPR = IPSC_2_/IPSC_1_) was used to estimate potential differences in synaptic release probability [31]. At the end of some experiments IPSCs were blocked with 10 μM Gabazine (n = 6; data not shown), to confirm the GABAergic nature of inhibitory currents.

To ensure that series resistance remained stable throughout the experiments, we analyzed current relaxation induced by a 10 mV step from the holding potential (0 mV and -70 mV for inhibitory and excitatory currents, respectively). Consistently with previous reports [20,34,35], transients were reliably fitted with a mono-exponential function yielding membrane capacitance of 2.6 ± 0.2 pF, input resistance of 1.8 ± 0.1 GΩ, and series resistance of 17.4 ± 0.5 MΩ (n = 25). These parameters were monitored during the perfusion of anesthetic and none of them was significantly changed by desflurane. Furthermore, “resting” membrane potential was monitored throughout current clamp recordings. Intrinsic excitability of GrCs was evaluated by measuring the amount of current injected to elicit at least one action potential from a membrane potential of -60mV, while spike after-hyperpolarization was measured as the difference between spike threshold and the minimum level of membrane potential after the spike.

### Perfusion with Anesthetics

Aqueous anesthetic solution was prepared to obtain a final concentration of 2 MAC (Minimum Alveolar Concentration) in the anesthetic reservoir. MAC is referred to volatile anesthetics and it provides the value of concentration at which half of the tested patients undergoes loss of consciousness or, alternatively, animals loss right reflex [36]. We employed a concentration of 2 MAC in the anesthetic reservoir for the following reasons: i) desflurane has a boiling point of about 24°C. In our experiments, desflurane was used in aqueous solution and perfused at 32° in an open system, thus accelerating the rate of dispersion from the recording chamber; ii) desflurane concentration has been typically used in a wide range (0.5–5 MAC) (e.g. [3,5,7]); iii) finally, the actual anesthetic concentration in the tissue depends on the molecule penetration which is affected by its hydrophobic nature. We therefore decided to work in the upper limit of concentration range to compensate for molecules dispersion.

The desired concentration was obtained by adding 6 ml of desflurane (Baxter, Deerfield, IL, USA) directly to the extracellular solution up to a total volume of 50 ml in a closed vial (4% vol/vol; 2 MAC). Vials were shaken and left 60 minutes to equilibrate before filtering (0.5μm diameter) and adding the supernatant directly to the gravity-driven perfusion system [37]. In some experiments (n = 4), actual anesthetic concentration in the recording chamber was determined by means of gas chromatography coupled with mass spectrometry (GC-MS). A GC 7890A (Agilent Technologies, Waldbronn, Germany), coupled with a single quadrupole 5975C TAD Series GC/MSD system (Agilent Technologies). Identification of desflurane was achieved by using mass fragmentation data and comparison with the literature. According to GC-MS quantification, the solution in the recording chamber contained desflurane at an aqueous concentration of 9.1±2.1 mM (mean±sd; n = 4; p<0.01).

### Mutual Information Estimation

Information theory can be used to estimate the amount of information transmitted by neurons. In this context, the correlation between a set of input stimuli and output responses allows to quantify the information conveyed by neurons [16]. In order to estimate the level of correlation, time windows of neuronal responses can be divided into temporal bins, to be further digitized in dependence on the presence of a spike (Fig 1B). The resulting sequence of temporal digits can be considered as a binary word. Neurons respond to input stimuli with a variety of binary words generating, as a whole, the neuronal vocabulary. The largest the vocabulary, the richest the conveyed information. Nevertheless, a reliable communication is ensured by a significant correlation between the output word and the input stimuli. The estimation of the correlation degree can be performed by calculating “*response entropy*” [13], which represents the variability of neuronal responses to a set of input stimuli. The largest this entropy the higher the capability of a system to communicate. Stochastic neurotransmitter release, non-linear integration mechanisms and spontaneous firing introduce variability in output responses when repeatedly presenting the same input. This, in turn, introduces a systematic error that needs to be accounted for and that can be calculate by “*noise entropy*” [13]. MI is obtained by subtracting “*noise entropy*” from “*response entropy*” and it quantifies how robust to noise is the information conveyed by neural activity [15]. Given these premises, MI is measured in bits and can be calculated through the following equation:

$$MI=\underset{s}{\sum }\underset{r}{\sum }p\left(s\right)p(r|s)\;{\text{log}}_{2}\frac{p(r|s)}{p\left(r\right)}$$

where *r* and *s* are the response pattern and the stimulus pattern, respectively, in terms of binary digits (see Fig 1B); *p*(*r*) is the probability that a response pattern (*r*) occurs within a single acquisition; *p*(*s*) is the probability that a stimulus pattern (*s*) occurs within a single acquisition; *p*(*r|s*) is the probability of having a pattern response (*r*) given a stimulus pattern (*s*).

The correct calculation of MI derives from an accurate estimation of probabilities to be inserted into the equation. Experimental limitations provoke fluctuations which, in turn, alter the estimation of probabilities. This bias can be only overcome by increasing the number of repetitions and by employing a system with a reduced variability of neuronal responses. The cerebellar granular layer microcircuit allows to obtain MI quantification between mf and GrCs because: i) mf inputs are conveyed through high-frequency bursts (up to 500Hz [38,39]) that have been well characterized, enabling a proper representation of the input space [30,40]; ii) in cerebellar slices GrCs are silent when mf are severed, reducing spontaneous firing and output variability; iii) in response to mf activation, GrCs generate only few spikes (typically less than 3–4) with a maximum output frequency of 100–150 Hz, reducing the output response space.

MI transfer was calculated by adopting an experimental strategy similar to the one reported in [23] and MI values, obtained in different pharmacological conditions, were compared.

GrCs responses were spike-sorted via a threshold-crossing procedure (Matlab; Mathworks inc. Oregon, USA). To compute MI, both input (mf) and output (GrC) spike trains were converted into binary digits (see Fig 1B). Mf spike trains were digitized by using a 10 ms temporal bin corresponding to a maximum input frequency of 100 Hz. Each stimulus pattern lasted for 40 ms (4 digits) generating an input set of 2^4^ = 16 stimuli (all possible combinations). Experimental limitations, due to the time required to explore the input set, prompted us to limit to 8 different stimuli the input space by excluding duplications (e.g., 1010 and 0101 were considered equivalent in terms of spike counting and probability of firing, while 0000 was not considered because unable to induce spikes). We have decided to adopt this reduced input space since mf typically discharge with bursts of 2–4 action potentials at an average frequency of 100 Hz [39].

GrCs responses were digitized by using a 6-ms bin width. This bin width was chosen since GrCs output frequency is typically lower than 150 Hz, therefore the probability of observing two spikes in the same bin was minimized. A 60-ms time window was used for sampling the GrC output (10 digits) since, in the presence of synaptic inhibition, GrCs rarely generate spikes beyond this time window. Experimental limitations prevented us to independently stimulate single excitatory afferent fibers; we thus used the same set of stimuli simultaneously on all the activated fibers.

Response variability to the same input was reduced by repeating each stimulus 25 times. This number appears particularly low if compared to the whole set of possible combinations of the output pattern (10 digit output code = 2^10^). Our approach in fact, (also known as “plugin estimator” [16]), has the potential of introducing biases related to single trial variability that could be reduced by adopting different experimental strategy (e.g., PT Bayesian estimation or NSB entropy estimator). None of these approaches enabling the bias correction were adopted. However mathematical modeling and simulations reported in [23] showed that a similar experimental protocol provides a good estimation of MI. In particular, this method was shown to be statistically significant when MI assumed values larger than 0.4 bits.

Beside MI calculation, neuronal communication was investigated by analyzing the following parameters: 1) the probability of eliciting spikes, which was calculated as the percentage of traces in which the stimulation was capable of eliciting at least one spike; 2) the total number of spikes; 3) the average 1^st^ spike delay; 4) the 1^st^ spike jitter, which was calculated as the standard deviation of the first spike latency from the beginning of the stimulation; 5) the firing frequency which was calculated from the time difference between two consecutives spikes when the train of stimuli elicited at least two spikes.

### Statistical Analysis

Data are reported as means ± standard error of the mean (SEM) and statistical comparisons are done using paired Student’s *t*-test.

## Results

### Desflurane Decreases mf-GrC Mutual Information

In the cerebellum granular layer, mf simultaneously excite GrCs and Golgi cells (GoCs), which, in turn, are activated by GrCs through feedback loops and inhibit GrCs through feedforward loops (Fig 1A). The impact of desflurane on the information transfer between mf and GrCs was evaluated by estimating changes of mutual information (MI).

MI can be estimated by evaluating the level of correlation between input and output. To this aim, neuronal responses were divided into temporal bins, which were digitized in dependence of the presence of a spike (see Materials and Methods). Current-clamp recordings from GrCs were performed while synaptically eliciting firing (see Fig 1B). Spike detection was organized to generate binary words, which were generated by sequences of binary digits (see Materials and Methods and Fig 1B). In order to calculate MI, the probability of observing the same pattern in response to each input stimuli was calculated and employed to generate a cumulative probability. This, in turn, was used to determine the level of correlation between input and output (see Materials and Methods). The estimation of mf-GrC MI yielded a value of 2.2 ± 0.1 bits (n = 11) in control conditions, in agreement with previous observations [23]. This value indicated the level of functional correlation between mf signals and GrCs responses for a particular set of stimuli. Desflurane altered GrCs firing patterns (Fig 1C) and caused a significant reduction of MI (-28.8 ± 3.2%, p < 0.01; n = 7; Fig 1C–1E), likely due to a decreased variability in action potential generation. These findings indicated that desflurane hampered the correlation between input and output, without abolishing neuronal firing. The perfusion of desflurane in the presence of GABA-A receptor blocker SR9519 (gabazine) throughout the recordings did not induce any significant change in the firing pattern nor in MI (+2.2 ± 4.1%, p > 0.4; n = 6; Fig 1D and 1E), indicating that GABA-A receptors played a crucial role in desflurane-induced modulation of neuronal communication.

**Fig 2:**
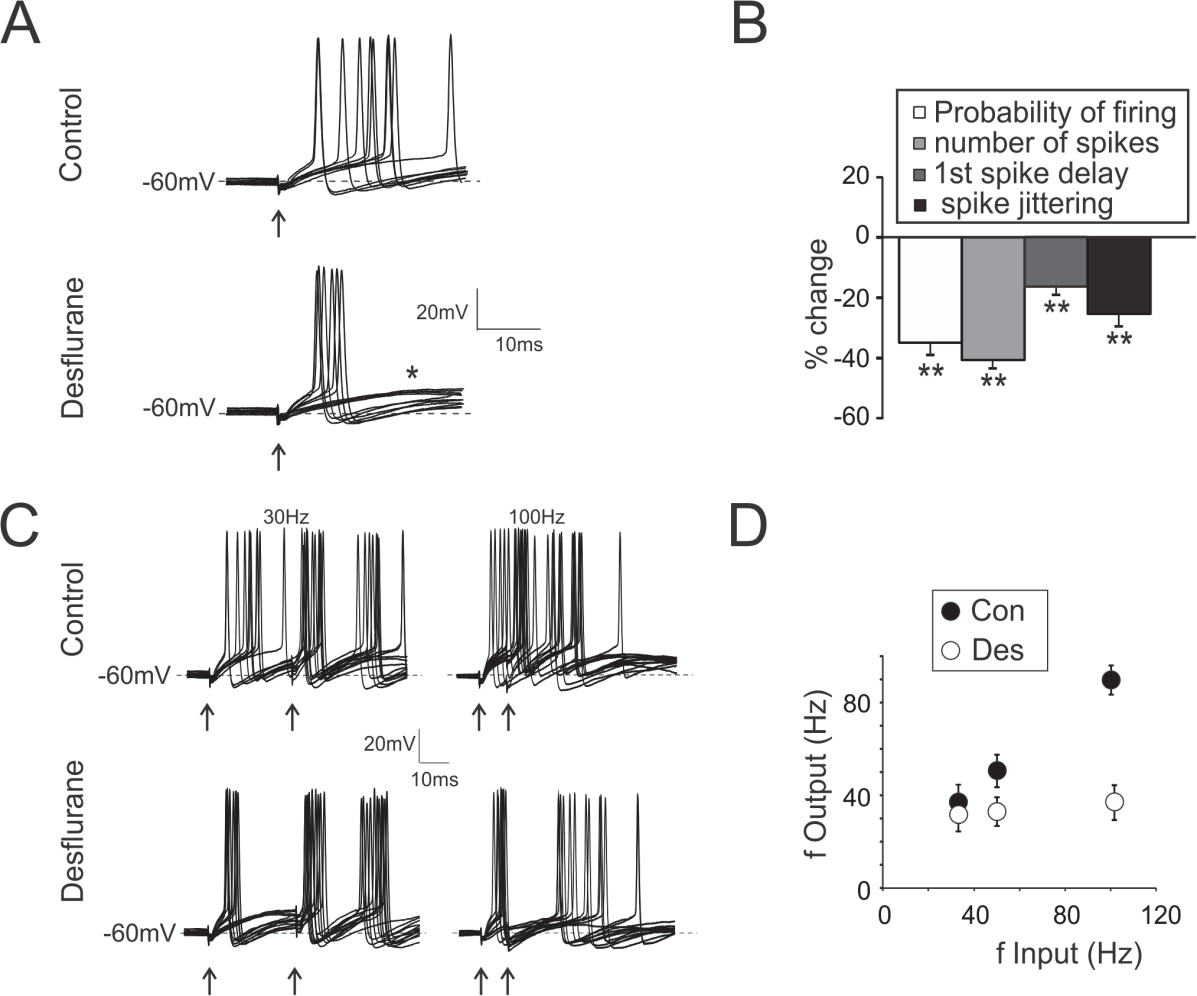
Modulation of granule cell firing by desflurane. **A.** Spikes elicited in response to mf stimulation (single pulse, arrow). Traces show 10 superimposed responses in control and during desflurane application. Note the decrease in the probability of eliciting spikes (increased failures: asterisk) and in the number of emitted spikes. Desflurane synchronizes firing as indicated by reduced spike jittering. **B.** Histogram summarizes variations in spike related parameters induced by deflurane. **C.** Spikes from GrCs elicited in response to pairs of stimuli at different frequencies (30–100 Hz, 15 superimposed traces). Desflurane decreases the probability of eliciting spike, the total number of emitted spikes and the firing frequency (n = 7). **D.** Relationship between input frequency (f Input: spikes in mf) and output frequency (f Output: spikes in GrCs) in control (Con) and during desflurane application (Des) (n = 7).

GrC firing properties were evaluated by applying supra-threshold single stimuli. Desflurane (Fig 2A) decreased the probability of synaptically eliciting spikes (-34.9 ± 4.1%, p < 10^−3^; n = 7; Fig 2A and 2B), as well as the total number of emitted spikes (-41.2 ± 3.6%, p < 10^−6^; n = 7; Fig 2A and 2B). The 1^st^ spike latency and its standard deviation were decreased (-17.1± 2.6%, p < 0.01 25.6 ± 4.1%, p < 0.01; respectively n = 7, Fig 2A and 2B).

Given the impact of desflurane on GrC firing pattern, we asked whether this anesthetic could affect the relationship between mf and GrC firing frequency, which, in control conditions, is quasi-linear [20,40,41] (Fig 2D). Desflurane abolished the dependency of the output from the input frequency (Fig 2C and 2D) and GrCs average firing frequency was markedly reduced when incoming mf bursts were delivered at 100 Hz (-52.3± 7.4% (n = 7, p < 0.01; Fig 2D).

In conclusion, desflurane did not abolish GrCs capability of eliciting spikes, but made firing more regular and insensitive to changes in input currents. The anesthetic converted spike induction from high to low probability while increasing spike output synchrony.

### Desflurane Increases GABA-A Receptors Activity in GrCs

**Fig 3:**
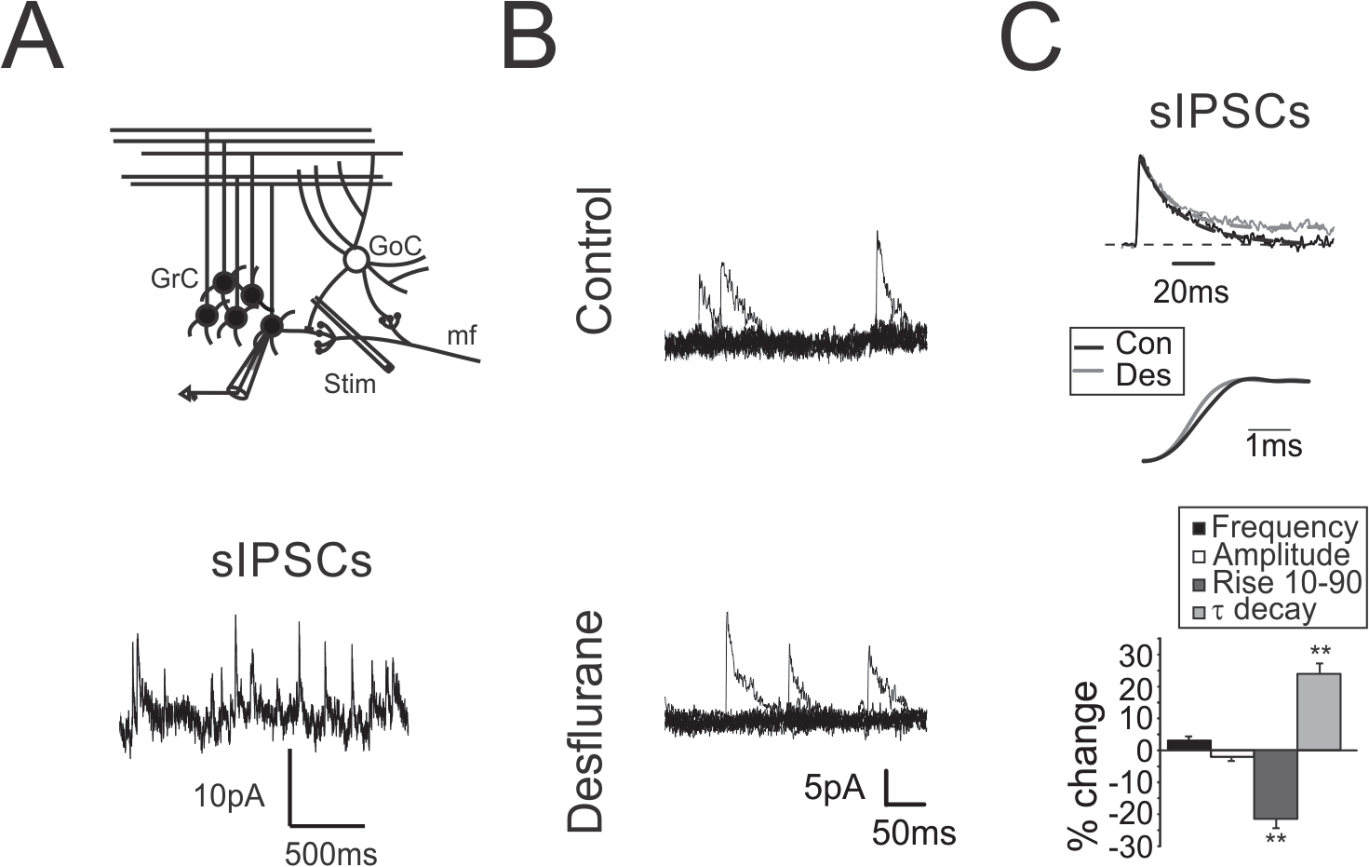
Modulation of spontaneous GABAergic inhibition by desflurane. **A.** Scheme of the granular layer microcircuit. The stimulating electrode (stim) is placed in the surrounding of the recorded GrC to evoke action potentials in the GoC axonal plexus. *Traces*: Spontaneous IPSCs recorded from GrCs voltage clamped at 0 mV reflects the autorhythmic discharge of GoCs. **B.** sIPSCs recorded from a granule cell before (top) and after (bottom) the application of desflurane. Note that sIPSC frequency and peak amplitude are unchanged. **C.** Normalized sIPSCs recorded from a GrC before (black) and during (gray) desflurane application. The mono-exponential fitting of the current relaxation reveals significant changes in the decay while the rise time is decreased (middle). Histogram summarizes the effects induced by desflurane on the biophysical properties of spontaneous inhibitory currents (n = 7).

**Fig 4:**
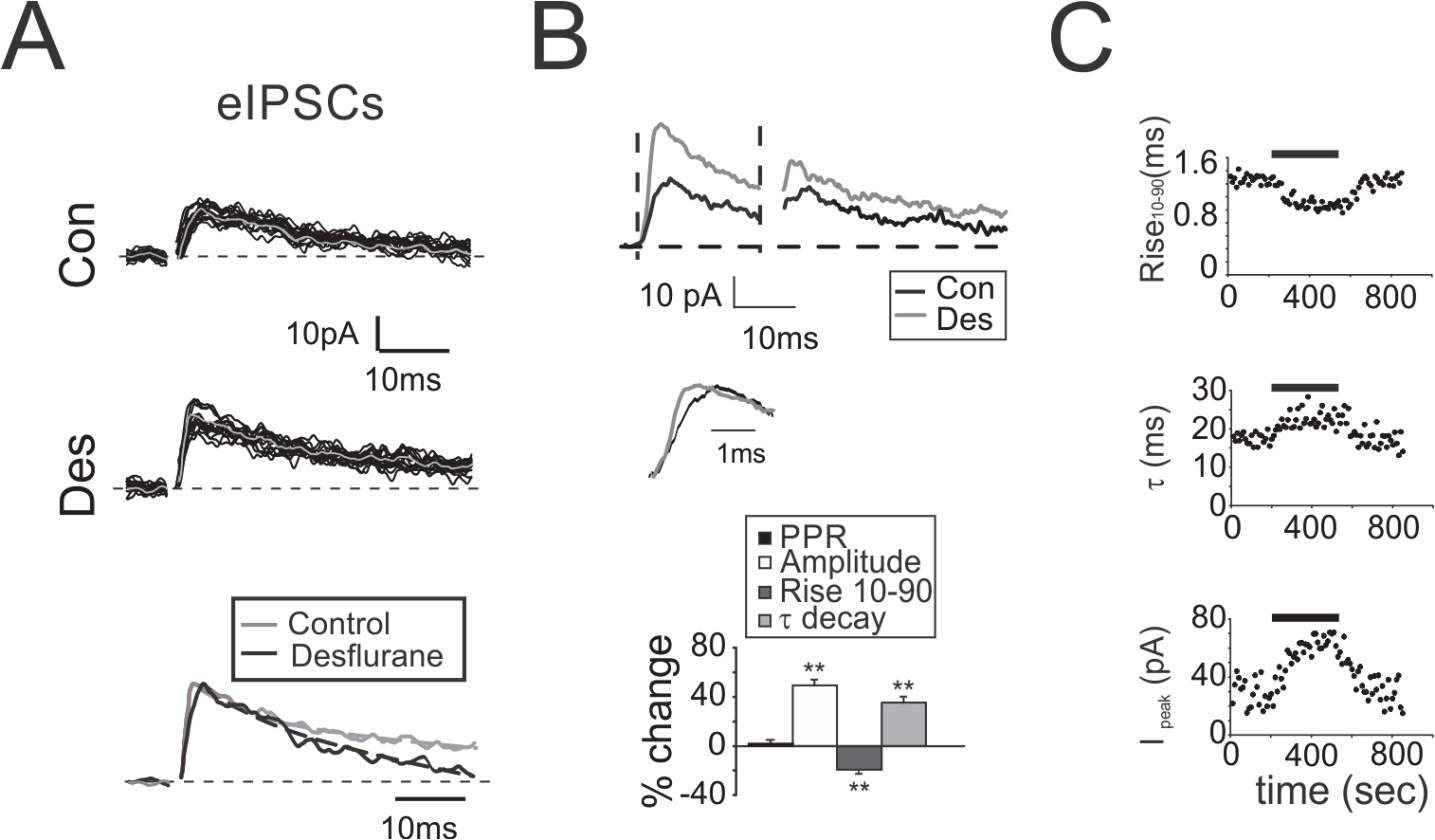
Modulation of evoked GABAergic inhibition by desflurane. **A.** Evoked IPSCs elicited by a single stimulus before (Con) and during desflurane (Des). Gray traces indicate the average of the 15 superimposed traces. *Bottom*: Normalized average traces taken from upper panels. Monoexponential fitting (dashed lines) show the decreased rise and the increased decay time **B.** eIPSCs elicited by a pair of stimuli at 50Hz recorded from a different granule cell before (black) and during (gray) desflurane perfusion. *Middle*: rise time. Histogram summarizes the variations induced by desflurane on eIPSC biophysical properties (n = 7). **C.** Time courses of the effect of desflurane (bar) on eIPSCs rise time (Rise_10–90_), decay time constant (τ) and peak amplitude (I_peak_). Note the rapid effect onset (less than 30 sec). Steady state is obtained in less than 100 seconds.

The increased GrCs firing regularity observed in the presence of desflurane was not confirmed when recordings were performed in the presence of gabazine (Fig 1D). We therefore investigated the effect of desflurane on GrC GABA receptors, which are controlled by synaptic inputs from Golgi cells (Fig 3A) [22]. Inhibitory currents were recorded in the presence of 20 μM NBQX and 50 μM D-APV, to block glutamatergic neurotransmission, and were identified as positive current deflections by voltage clamping GrCs at 0 mV. GoCs are spontaneously active [42,43]: thus, both spontaneous inhibitory post-synaptic currents (sIPSCs; Fig 3A and 3B) and evoked IPSCs (eIPSCs; Fig 4A) induced by electrically stimulation of GoCs axons were detected and analyzed. sIPSCs occurred at an average frequency of 2.8 ± 0.5 Hz (Fig 3A bottom, n = 11) in agreement with previous observations [42,43]. sIPSCs were abolished together with eIPSCs by 10 μM gabazine (n = 6; data not shown) confirming their GABergic origin. sIPSCs and eIPSCs shared similar kinetics both for rise time (rise_10–90_) (sIPSC: 1.41 ± 0.1 ms, n = 11; eIPSCs: 1.39 ± 0.1 ms, n = 11) and for decay time constant (τ) (sIPSCs: 21.4 ± 0.8 ms; n = 11; eIPSCs: 23.1 ± 1.1 ms; n = 11), indicating that GABAergic currents elicited by electrical stimulation were similar to the ones spontaneously activated by Golgi cells.

The occurrence of sIPSCs as well as their peak amplitude was unchanged during desflurane perfusion (Fig 3B and 3C histogram), whereas rise_10–90_ was decreased (-21.3± 3.1% Fig 3C traces and histogram; n = 7; p<10^−4^) and τ was increased (+24.6± 2.6% Fig 3C traces and histogram; n = 7; p<10^−5^ Fig 3C traces, histogram). These results confirmed that desflurane, similarly to other halogenated compounds [44,45], interacted with GABA-A receptors potentiating total charge transfer evaluated as IPSC area (+40.3 ± 6.3%, n = 11; p < 10^−4^; [33]).

The analysis of eIPSCs kinetics confirmed the observations on spontaneous currents, namely, a reduction of the rise_10–90_ (-19.2 ± 2.2%, n = 7; p < 10^−2^; Fig 4A and 4B histogram) and an increase for τ (+36.1 ± 5.7%, n = 7; p < 10^−2^; Fig 4A and 4B histogram) along with an increase in the total charge transfer (+84.7 ± 10.5%, n = 7; p < 10^−5^; Fig 4A and 4B histogram). Surprisingly, peak amplitude of eIPSCs was markedly increased (+48.6 ± 7.4%, n = 7; p < 0.01; Fig 4A and 4B histogram) suggesting either a change in presynaptic release mechanism or a variation in the intrinsic excitability of presynaptic neuron (GoC). Since both sIPSC amplitudes (Fig 3C histogram) and PPR did not change (+0.6 ± 1.5%, n = 7; p >0.2; Fig 4B histogram), an effect onto the release machinery was unlikely.

One concern when performing *in vitro* experiments with volatile anesthetics such as desflurane is related to the actual anesthetic concentration and to the time required to reach the steady state effect at the action site. The time course of the effect of desflurane was evaluated by recording single eIPSCs elicited every 10 sec. Effects on rise time, decay, and amplitude were evident in less than 30 seconds and reached the steady state in about 100 seconds (Fig 4C).

### Desflurane and Excitatory Neurotransmission on GrCs

In order to estimate the impact of desflurane onto GrCs excitatory neurotransmission, mf were stimulated and evoked excitatory post-synaptic currents (eEPSCs) were recorded by voltage clampling GrCs at -70 mV. eEPSCs were characterized by the typical glutamatergic waveform [46] comprising a rapid AMPA and slow NMDA component, as previously described [35].

**Fig 5:**
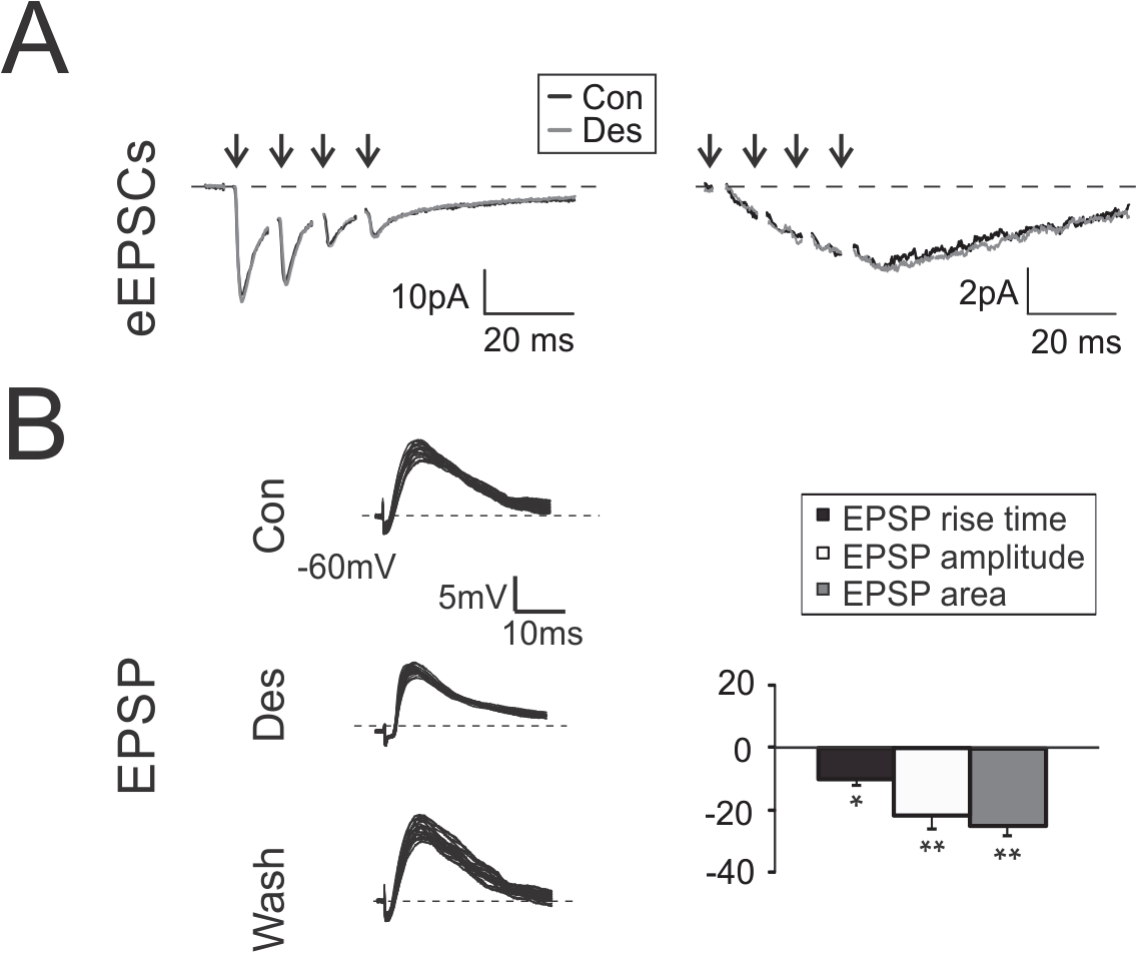
Modulation of excitatory neurotransmission by desflurane. **A.** *Left*. Evoked EPSCs elicited in response to 4 pulses at 100 Hz and recorded from a GrC voltage clamped at -70 mV in control (black) and during (gray) desflurane perfusion. *Right*. EPSC elicited from a GrC voltage clamped at -40 mV in the presence of gabazine and NBQX, in control (black) and during (gray) desflurane perfusion. **B.** EPSPs elicited by sub-threshold stimuli in GrC at -60 mV (20 superimposed traces). Histogram summarizes the effects of desflurane perfusion on EPSPs (n = 7).

In response to a 4-pulse, 100-Hz burst, the rapid eEPSCs component showed the typical short-term depression pattern [31], which was not affected by the application of desflurane (1^st^ peak amplitude change +7.2 ± 2.3%, p > 0.3; n = 8, Fig 5A left traces). This evidence indicated that AMPA glutamate currents were not targeted by desflurane, as shown for other halogenated anesthetics [1]. In addition, desflurane did not change the residual, slow component of glutamatergic currents (Fig 5A left traces), suggesting that NMDA receptor activity was not altered by the anesthetic. To further investigate the impact of desflurane on NMDA currents, which are known to be affected by some halogenated anesthetics, eEPSCs were recorded from GrCs voltage clamped at -40 mV (Fig 5A, right traces) and in the presence of 10 μM gabazine and 10 μM NBQX, a selective AMPA receptor blocker, to better isolate NMDA component. The typical NMDA current showed a marked temporal summation peaking at 10–20 ms from the last stimulus (-3.8±0.2 pA, n = 4 at the peak of the current, Fig 5A, right traces). NMDA peak amplitude and current kinetics were not altered by desflurane (+0.9±1.8%, n = 4; p>0.4, Fig 5A right traces), differently from other halogenated compounds [47,48].

The effect of desflurane on excitatory neurotransmission was further evaluated by recording excitatory post-synaptic potentials (EPSPs) elicited by mossy fibers stimulation. In this configuration feed-forward GABAergic inhibition activated with a di-synaptic pathway is free to block membrane depolarization with an effect proportional to the amount of depolarization [49]. Desflurane rapidly and transiently modified EPSPs kinetics and amplitude (Fig 5B), by reducing EPSP rising phase (-11.9±1.4%, n = 7, p<0.05 Fig 5B), peak amplitude (-21.2±3.4%, n = 7 p<10^−3^ Fig 5B) as well as total depolarization evaluated as EPSP area (-24.4±2.1%, n = 7 p<10^−4^ Fig 5B). Excitatory neurotransmission was not affected by desflurane, as confirmed by the fact that EPSPs recorded in the presence of gabazine were unchanged by desflurane perfusion (n = 4; data not shown). Thus, reduction in the EPSP total depolarization and peak amplitude could be entirely attributed to a potentiated synaptic inhibition, which affected membrane potential depending on the level of depolarization. Reduction in EPSP rising phase arose likely from post-synaptic membrane potential integration mechanisms.

### Desflurane Increases GrC Intrinsic Excitability

**Fig 6:**
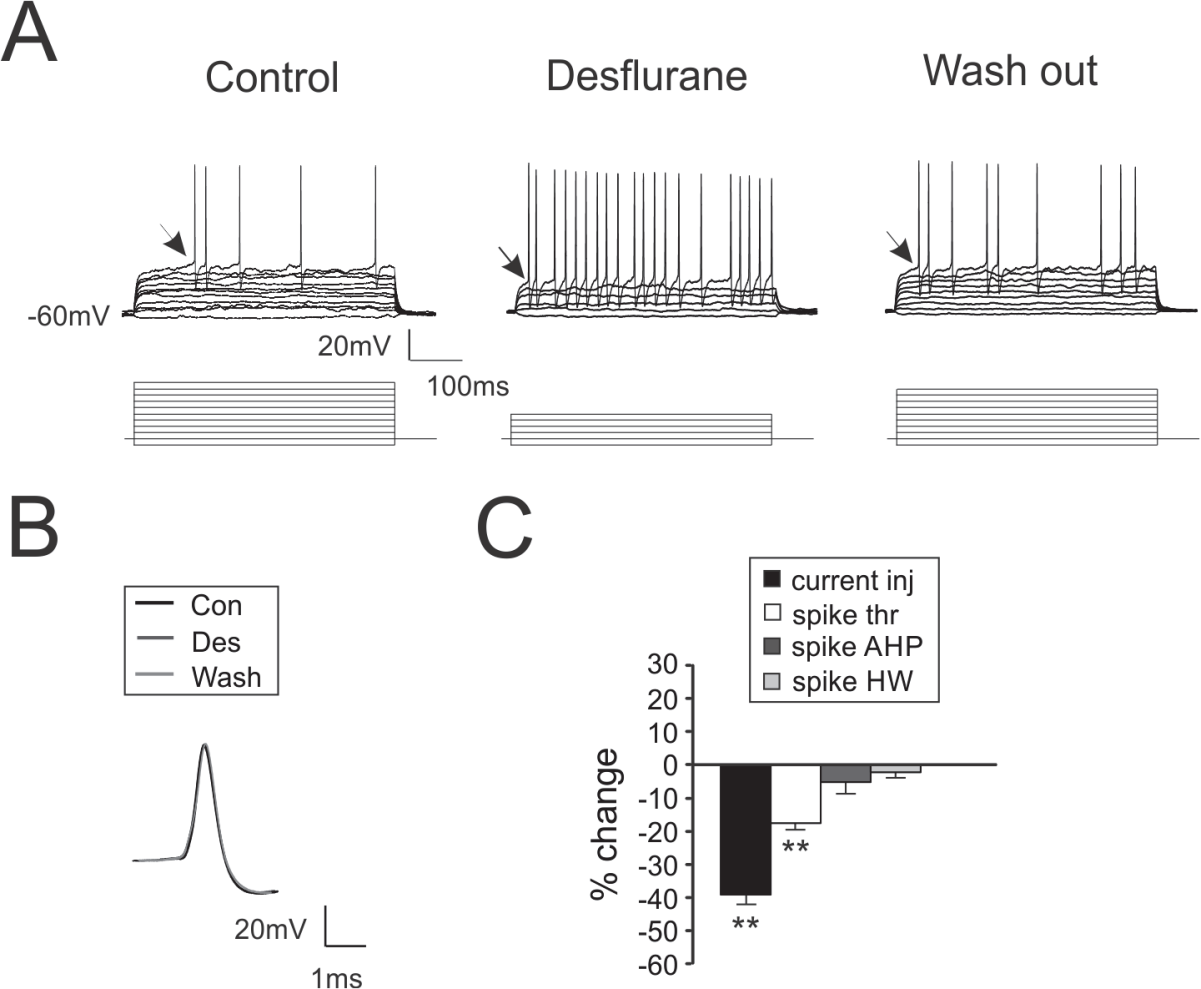
Desflurane increases intrinsic excitability of granule cells. **A.** GrC voltage responses to current injections (bottom traces 1 pA/step) in control conditions, during desflurane perfusion and following wash-out. Note the decreased number of current steps required to generate action potentials and the increased number of elicited spikes, concomitant with a reduced firing threshold (arrow). **B.** Comparison of spike waveform obtained in control condition (black), in the presence of desflurane (dark gray) and following wash-out (light gray). **C.** Histogram summarizes the variations induced by desflurane on the current needed to elicit spikes (current inj), spike threshold (spike thr), spike after hyperpolarization (spike AHP) and spike half-width (spike HW) (n = 7).

Whole-cell current-clamp recordings from GrCs were performed to investigate whether desflurane affected GrCs intrinsic excitability. The zero-current potential (a rough estimation of resting membrane potential in patch clamp experiments [50]) was monitored throughout the recordings: no significant variations could be observed during desflurane perfusion (data not shown). In the presence of desflurane, current needed to generate action potentials was significantly reduced (-39.9 ± 4.1%, p < 10^−4^, n = 7; Fig 6A–6C) by virtue of a spike threshold decrease (from -44.6 ± 0.7 mV to -53.4 ± 1.6 mV n = 7; Fig 6A–6C). Membrane resistance did not change, as indicated by input resistance measurement (passive ΔV/ΔI; Fig 6A) as well as the zero-current potential (data not shown). Initial excitability recovered upon wash-out (-5.9 ± 3.1% p>0.1, n = 7). Finally, half-width and after-hyperpolarization of the action potential were not altered by desflurane perfusion (Fig 6B and 6C). Thus, desflurane increased the intrinsic excitability of GrCs, that is, these cells were more prompt to generate action potentials, as suggested also by the decreased EPSP rise time (Fig 5B) and1 ^st^ spike delay (Fig 1A and 1B).

As a whole, these data indicated that desflurane had opposing effects on cerebellar GrCs bringing to the observed reduction in MI: intrinsic excitability of GrCs was increased by virtue of a lowered spike threshold, while synaptic excitability was decreased reflecting a potentiation of GABAergic inhibition, making neuronal firing more regular and stereotyped.

## Discussion

This paper shows that desflurane, by potentiating synaptic inhibition and post-synaptic excitability, synchronizes spike activity and reduces variability of action potential generation in a central synapse. This, in turn, leads to a global reduction of the amount of information to be transferred among neurons rather than silencing neuronal communication in a non-specific manner. These results suggest that mechanisms exploited by desflurane on neuronal circuits leads to a more regular neurotransmission [51], which, however, is less rich of information.

### Modulation of MI by Desflurane

The concept of mutual information was introduced in Neuroscience to evaluate the capability of a neuronal circuit to transfer information [8,52] and to estimate how much neuronal networks can integrate information coming from different circuits or from different temporal input patterns [53]. Here we investigated the alterations induced by desflurane on neuronal communication by adopting a simplified set of physiologically relevant stimuli rather than extensively exploring the entire input space. This experimental strategy prevented us to univocally determining the absolute value of MI at the synapse between mf and cerebellar GrC [9,10,23]. However, it allowed us to quantitatively estimating the way neuronal communication in a central circuit is modulated by a general anesthetic. The choice of input set and the variability of output responses are the most important factors to obtain meaningful MI values.

Mf-granule cell synapse in acute slice is a suitable preparation for such purpose because of the following morphological and physiological factors: i) spontaneous glutamate release from mf rarely occur [31,54] and can hardly elicit action potentials in GrCs. This limits background noise and reduces stochastic variability. ii) Although during proprioceptive or vestibular activation mf respond with tonic, low frequency firing [55,56], sensory activation elicit high-frequency bursts discharge (average frequency 100 Hz [39,57]). The employed protocol therefore reproduced a realistic pattern occurring *in vivo*, and this pattern was preferred to low frequency firing, because the temporal window to be sampled could be significantly reduced. iii) GrCs response patterns to mf bursts are stereotyped, last for few tens of milliseconds and have been well characterized. iv) A similar experimental approach was validated by mathematical simulations attesting the significance of the protocol (see [23]). Such investigation can be hardly carried out in other neuronal preparation in which output or input variability is much higher.

The marked decrease of MI induced by desflurane can be attributed to spike synchronization, which, in turn, is a consequence of concomitant effects on post-synaptic excitability and on synaptic inhibition. The processing of input signals in the granular layer typically occurs at the millisecond time scale [39,58]. In fact, the high-fidelity exhibited by GrCs in converting mf inputs [39] allows these neurons to faithfully analyzing incoming information by decoding first spikes timing [58]. Moreover, the intervention of GABAergic inhibition with a few ms delay, hampers the temporal summation of different incoming signals, implementing time windowing operations [59]. The reciprocal interactions between excitation and inhibition occurring in narrow time-windows make GrCs fine detectors of coincidence events brought by incoming signals [21,46,60]. Desflurane, by decreasing the rise time of inhibitory currents (Fig 4B), narrowed the temporal window which permits spike emission (Fig 2A and 5B). In addition, the increased GrCs excitability induced by desflurane, further anticipated action potential emission (see Fig 2A) even though neurotransmission fidelity was decreased (increased spike failures Fig 2A). Timing of inhibitory loops onto excitatory inputs was affected by the altered kinetics of GABAergic currents, as evidenced by longer decays of inhibitory responses (Fig 4A). The suppression of input-output dependency and the limited maximum emitted frequency (Fig 2D), could in fact be explained by a stronger and prolonged action of inhibitory neurotransmission (Fig 4A and 4B). As a whole, these results suggest that desflurane enhances regularity in excitatory neurotransmission by increasing the strength of synaptic inhibition.

### Cellular Mechanisms Underlying the Effect of Desflurane on MI

In GrCs, phasic and tonic GABAergic currents [22,61] regulates repetitive discharge [46,62] and excitability, respectively [63]. The increased GrC intrinsic excitability indicates that desflurane exploits its action mainly through the regulation of phasic mechanisms triggered by mf discharges [64], since an increase of tonic inhibition would have the opposite effect. In addition, the potentiation of current decay induced by desflurane increases temporal summation of inhibition rather than uniformly suppressing GrCs excitation. This potentiation alters short-term plasticity mechanisms occurring in GrCs GABAergic synapses in response to repetitive activation. Finally, recurrent feedback activation [65] and local controls taking place in the glomerulus [66] could further reinforce these mechanisms.

Like other halogenated compounds [4,33,67], desflurane slows down GABAergic current decay and increases the total charge transfer. Surprisingly, this anesthetic also accelerated rise time, suggesting a modulation of the kinetics in the anesthetics-receptor complex [5,68].

Our results indicate that desflurane did not alter GrCs glutamatergic currents (Fig 5A), suggesting that the marked reduction of depolarizing responses was entirely related to inhibitory activity rather than to a pure decrease in the excitatory strength. The effect of desflurane on spike threshold strongly points to a modulation of sodium channel gating, possibly involving a hyperpolarizing shift in the voltage dependence of activation [69]. In particular, the increased GrCs excitability could originate from changes in the sodium persistent current (INa_p_), where the small amplitude of this conductance has a large impact on membrane excitability [35,70,71]. It cannot be excluded that the increased GrCs excitability could also derive from the block of voltage-dependent potassium channels, in particular Kv3 and Kv1.1, largely expressed in the cerebellum [72], and whose gating was shown to be affected by desflurane.

### Volatile anesthetics and “in vitro” preparation

A critical point to be considered when dealing with volatile anesthetics and “*in vitro”* experiments concerns the proper range of concentration to employ (for details see [73] and [74]). It is widely accepted that only clinically adopted concentrations (0.2–2 MAC) are relevant for “*in vitro*” studies [73]. Effects induced by concentrations above the clinical range has been object of debate [74]. We decided to work at an initial aqueous concentration of 2 MAC for: i) the penetration in a dense and highly compact neuronal tissue such as the cerebellum granular layer is unknown and hard to be estimated; ii) the boiling point of desflurane (24°) is well below the temperature used during recording sessions (32°). An exact estimation of evaporation degree from the open recording chamber is unfeasible and it was not possible to assess the actual amount of anesthetics penetrating in the tissue; iii) the aim of our experiments was to observe relevant effects on physiological parameters related to neuronal activity.

Further investigations will be required to establish the correlation, if any, between anesthetic concentrations and changes in neuronal communication.

### Clinical implications and conclusions

Clinical and pre-clinical investigations by means of large scale techniques such as EEG and fMRI, have shown that general anesthesia could act by decreasing the complexity level of an integrated system [75] and could also reduce the number of functional states in a neuronal network [1,53]. However, integrative approaches do not unveil the cellular details underlying the alterations induced by anesthetics on network functioning. The mf-GrC synapse was employed as an experimental model for its relatively simple morphological architecture and the well characterized electrophysiological properties of granular neurons allow one to quantify relevant parameters (e.g., MI) that may shed light on the mechanisms controlling interactions between anesthetics and neurons.

Although the cerebellum is not classically considered as a main target of anesthetics for achieving clinical outcomes, recent experimental findings revealed that cerebellar functions are severely altered during general anesthesia [24–27]. Understanding the interactions between halogenated anesthetics, especially desflurane that shows the most favorable recovery profile [76], and cerebellum, which is central to most movement-related functions, may lead to an improved perioperative management and early mobilization.

The fragile balance between excitation and inhibition in subjects predisposed to epilepsia could be disrupted by the increased intrinsic excitability observed in the presence of desflurane leading to the emergence of epileptic seizures, especially in the pediatric and adolescent populations. One of the major concerns when providing anesthesia to a patient with epilepsy is in fact the tendency of general anesthetics to modulate seizure activity and to interact with antiepileptic drugs [77]. The clinical intervention is normally not required in healthy patients probably due to the correct balancing between neuronal excitability and synaptic inhibition. Desflurane, by increasing neuronal excitability could also contribute to determine side effects such as delirium and agitation observed during recovery from anesthesia especially in the pediatric population [78] where GABAergic currents might have depolarizing effects generating hyperexcitatory behaviors [79].

In conclusion, a better understanding of cellular mechanisms regulating neuronal communication and its modulation by halogenated compounds may lead to a fine titration of the level of anesthesia, opening promising perspectives on interventions during clinical treatments.
